# 4-Chloro­butyl 7-chloro-1-cyclo­propyl-4-(1,3-diethyl-4,6-dioxo-2-sulfanyl­idene-1,3-diazinan-5-yl­idene)-6-fluoro-1,4-di­hydro­quinoline-3-carboxyl­ate

**DOI:** 10.1107/S1600536813016024

**Published:** 2013-07-03

**Authors:** Kamal Sweidan, Salim F. Haddad, Murad A. AlDamen, Ahmed Al-Sheikhb

**Affiliations:** a Department of Chemistry, The University of Jordan, Amman 11942, Jordan; b Faculty of Pharmacy, Umm Al-Qura University, Makkah, Saudi Arabia

## Abstract

The title compound, C_25_H_26_Cl_2_FN_3_O_4_S, contains two bio-active moieties (thio­barbituric acid and fluoro­quinolone). In the crystal, mol­ecules are linked *via* C—H⋯O and C—H⋯F hydrogen bonds, forming two-dimensional slab-like networks lying parallel to the *bc* plane. The benzene ring substituted by F and Cl atoms and the 4-chloro­butyl group seem to be partly disordered, however attempts to model the disorder were unsuccessful.

## Related literature

For the biological activity of fluoro­quinolone derivatives, see: Li *et al.* (2000 ▶); Baker *et al.* (2004 ▶); Mitscher (2005 ▶). For the crystal structures of some fluoro­quinolone and 1,3-diethyl-2-thio­barbituric acid derivatives, see: Al-Qawasmeh (2012 ▶); Sweidan *et al.* (2012 ▶); Shishkin *et al.* (1997 ▶).
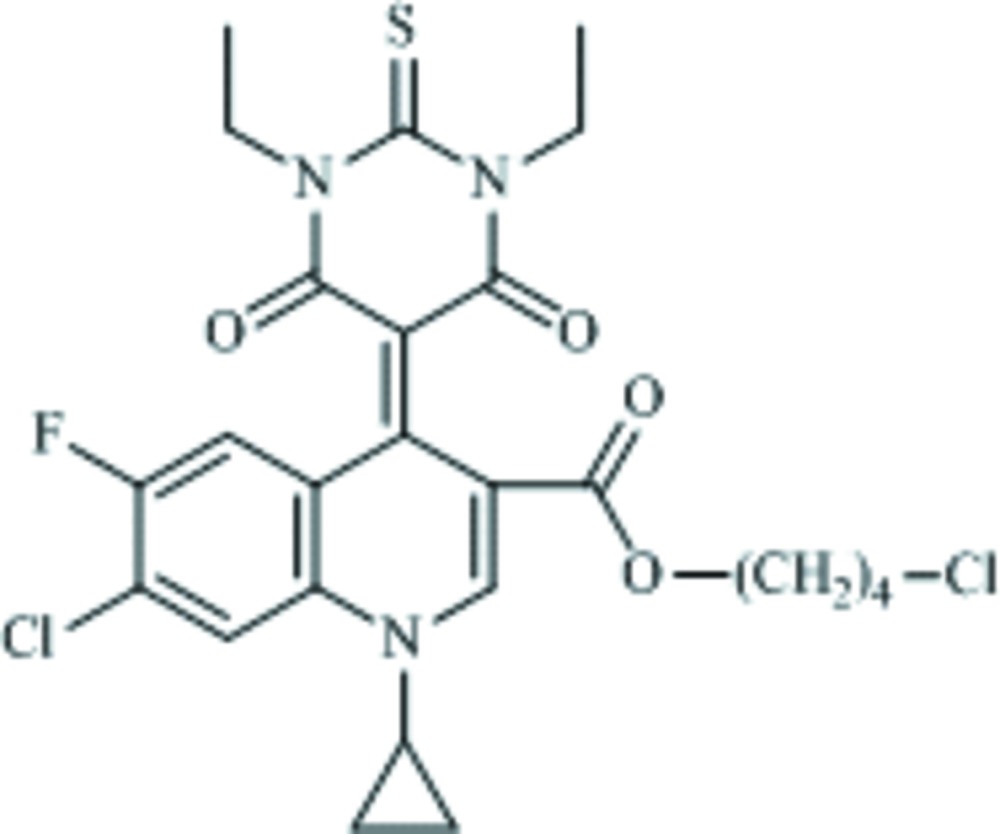


## Experimental

### 

#### Crystal data

C_25_H_26_Cl_2_FN_3_O_4_S
*M* *_r_* = 554.45
Monoclinic, 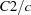

*a* = 24.3035 (15) Å
*b* = 13.8310 (8) Å
*c* = 16.4507 (16) Å
β = 107.345 (8)°
*V* = 5278.3 (7) Å^3^
*Z* = 8
Mo *K*α radiation
μ = 0.37 mm^−1^
*T* = 293 K
0.30 × 0.15 × 0.06 mm

#### Data collection

Agilent Xcalibur Eos diffractometer
Absorption correction: multi-scan (*CrysAlis PRO*; Agilent, 2011 ▶) *T*_min_ = 0.606, *T*_max_ = 1.000
12511 measured reflections
4664 independent reflections
2477 reflections with *I* > 2σ(*I*)
*R*_int_ = 0.047

#### Refinement

*R*[*F* ^2^ > 2σ(*F* ^2^)] = 0.078
*wR*(*F* ^2^) = 0.230
*S* = 1.02
4664 reflections
325 parameters
H-atom parameters constrained
Δρ_max_ = 0.58 e Å^−3^
Δρ_min_ = −0.40 e Å^−3^

### 

Data collection: *CrysAlis PRO* (Agilent, 2011 ▶); cell refinement: *CrysAlis PRO*; data reduction: *CrysAlis PRO*; program(s) used to solve structure: *SHELXS97* (Sheldrick, 2008 ▶); program(s) used to refine structure: *SHELXL97* (Sheldrick, 2008 ▶); molecular graphics: *ORTEPIII* (Burnett & Johnson, 1996 ▶); software used to prepare material for publication: *SHELXL97*.

## Supplementary Material

Crystal structure: contains datablock(s) I, global. DOI: 10.1107/S1600536813016024/bh2478sup1.cif

Structure factors: contains datablock(s) I. DOI: 10.1107/S1600536813016024/bh2478Isup2.hkl

Supplementary material file. DOI: 10.1107/S1600536813016024/bh2478Isup3.cml

Additional supplementary materials:  crystallographic information; 3D view; checkCIF report

## Figures and Tables

**Table 1:** Hydrogen-bond geometry (Å, °)

*D*—H⋯*A*	*D*—H	H⋯*A*	*D*⋯*A*	*D*—H⋯*A*
C18—H18*A*⋯O2^i^	0.98	2.32	3.014 (6)	127
C20—H20*A*⋯O3^ii^	0.97	2.56	3.233 (6)	126
C25—H25*A*⋯F1^iii^	0.97	2.51	3.371 (10)	148

Symmetry codes: (i) 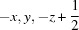; (ii) 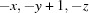; (iii) 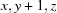.

**Fig. 1.**
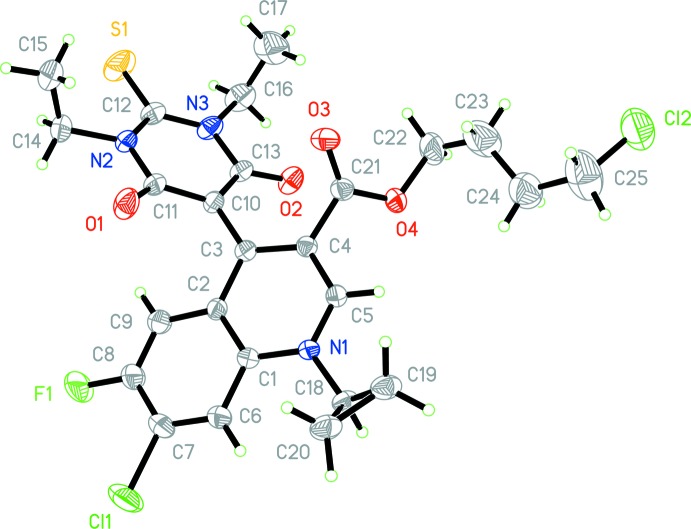
ORTEP drawing showing the molecular conformation of the title compound. Displacement ellipsoids for non-H atoms are drawn at the 30% probability level. Hydrogen atoms are represented as small spheres of arbitrary radii.

## supplementary crystallographic information

### Comment

A vast number of fluoroquinolones (Baker *et al.*, 2004) such as ciprofloxacin and moxifloxacin have therapeutic efficacy as anti-infective agents (Li *et al.*, 2000; Mitscher, 2005). The prepared molecule contains two bio-active moieties (thiobarbituric acid and fluoroquinolone), which may have a high impact biological effect.

From the X-ray structural analysis, it is interesting to note that this structure presents some zwitterionic character, since the bond length for C3—C10 is 1.449 (5) Å, intermediate between carbon-carbon single bond and double bond, while bond angles around C10 are close to 120°: C13—C10—C11 = 119.8 (4), C11—C10—C3 = 120.5 (4), and C13—C10—C3 = 119.5 (4)°. The crystal structure reveals the negative charge to be localized at the thiobarbituric-acid ring, with a contribution of the enolate form [C10—C11 = 1.418 (6), C10—C13 = 1.406 (6), C11—O1 = 1.231 (5), C13—O2 = 1.229 (5) Å; see Sweidan *et al.*, 2012]. The carbon-sulfur bond length is 1.665 (5) Å, which is close to that observed in a 2-thioxo-1,2,3,4-tetrahydropyrimidine derivative [1.673 (9) Å, Shishkin *et al.*, 1997; see also Al-Qawasmeh, 2012].

The displacement parameters in the 4-chlorobutyl branch are rather high, indicating, together with residual electron density in the vicinity, some degree of disorder. For example, C22—C23 bond length, 1.402 (9) Å, is a bit short for a single C—C bond. After convergence, it seems that benzene ring substituted by F1 and Cl1 could also be partly disordered. However, attempts to model such disordered parts did not improve the picture.

### Experimental

The title compound was prepared as followed: 8 ml of thionyl chloride was added to a solution containing 1.5 g (5.3 mmol) of 7-chloro-1-cyclopropyl-6-fluoro-4-oxo-1,4-dihydroquinoline-3-carboxylic acid in 15 ml of dry THF. The resulting solution was refluxed for 6 h, then cooled, and evaporated under reduced pressure to remove the excess thionyl chloride. To the resulting residue, a solution of 1,3-diethyl-2-thiobarbituric acid (1.19 g, 6.0 mmol) and 1.5 ml (10.0 mmol) of dry triethylamine in 20 ml of dry THF were added, at room temperature. After stirring overnight, the resulting precipitate was filtered off, washed with H_2_O/THF (1:2), and dried under reduced pressure. Yield: 1.5 g (51%). This solid was recrystallized from dichloromethane/diethylether, affording brownish crystals. A flat elongated crystal was mounted and data collected using five omega scans and a total of 277 frames with an exposure time of 76 s per frame.

### Refinement

All non-H atoms were refined anisotropically. H atoms were positioned geometrically, with C—H = 0.93 (aromatic CH), 0.96 (methyl CH_3_), 0.97 (methylene CH_2_), or 0.98 Å (methine CH), and constrained to ride on their parent atoms, with *U*_iso_(H) = 1.5*U*_eq_(carrier atoms) for methyl groups and 1.2*U*_eq_(carrier atoms) otherwise. Highest difference peak and hole in the last difference map are 0.58 and -0.40 e/Å^3^, respectively.

### Crystal data

**Table:**

C_25_H_26_Cl_2_FN_3_O_4_S	*F*(000) = 2304
*M**_r_* = 554.45	*D*_x_ = 1.395 Mg m^−^^3^
Monoclinic, *C*2/*c*	Mo *K*α radiation, λ = 0.71070 Å
Hall symbol: -C 2yc	Cell parameters from 2598 reflections
*a* = 24.3035 (15) Å	θ = 2.9–29.0°
*b* = 13.8310 (8) Å	µ = 0.37 mm^−^^1^
*c* = 16.4507 (16) Å	*T* = 293 K
β = 107.345 (8)°	Parallelpiped, orange
*V* = 5278.3 (7) Å^3^	0.30 × 0.15 × 0.06 mm
*Z* = 8	

### Data collection

**Table:**

Agilent Xcalibur Eos diffractometer	4664 independent reflections
Radiation source: Enhance (Mo) X-ray Source	2477 reflections with *I* > 2σ(*I*)
Graphite monochromator	*R*_int_ = 0.047
Detector resolution: 16.0534 pixels mm^-1^	θ_max_ = 25.0°, θ_min_ = 2.9°
ω scans	*h* = −28→22
Absorption correction: multi-scan (*CrysAlis PRO*; Agilent, 2011)	*k* = −16→16
*T*_min_ = 0.606, *T*_max_ = 1.000	*l* = −19→18
12511 measured reflections	

### Refinement

**Table:**

Refinement on *F*^2^	Primary atom site location: structure-invariant direct methods
Least-squares matrix: full	Secondary atom site location: difference Fourier map
*R*[*F*^2^ > 2σ(*F*^2^)] = 0.078	Hydrogen site location: inferred from neighbouring sites
*wR*(*F*^2^) = 0.230	H-atom parameters constrained
*S* = 1.02	*w* = 1/[σ^2^(*F*_o_^2^) + (0.1011*P*)^2^ + 5.5588*P*] where *P* = (*F*_o_^2^ + 2*F*_c_^2^)/3
4664 reflections	(Δ/σ)_max_ < 0.001
325 parameters	Δρ_max_ = 0.58 e Å^−^^3^
0 restraints	Δρ_min_ = −0.40 e Å^−^^3^
0 constraints	

### Fractional atomic coordinates and isotropic or equivalent isotropic displacement parameters (Å^2^)

**Table:**

	*x*	*y*	*z*	*U*_iso_*/*U*_eq_	
S1	0.24101 (6)	0.17120 (14)	0.14821 (12)	0.0943 (6)	
Cl1	−0.19348 (8)	0.14374 (13)	0.09519 (18)	0.1377 (10)	
Cl2	0.08427 (9)	1.00624 (14)	0.18342 (15)	0.1267 (8)	
F1	−0.07857 (15)	0.0682 (2)	0.1166 (3)	0.1128 (14)	
O1	0.03572 (14)	0.2291 (3)	0.0014 (2)	0.0712 (11)	
O2	0.12679 (13)	0.4217 (2)	0.2354 (2)	0.0586 (9)	
O3	0.08993 (13)	0.5366 (2)	0.0784 (2)	0.0578 (9)	
O4	0.06525 (13)	0.6089 (2)	0.1858 (2)	0.0612 (10)	
N1	−0.08816 (13)	0.4624 (2)	0.1034 (2)	0.0369 (8)	
N2	0.12960 (16)	0.2031 (3)	0.0780 (2)	0.0483 (10)	
N3	0.17566 (15)	0.3066 (3)	0.1881 (2)	0.0531 (11)	
C1	−0.08558 (17)	0.3631 (3)	0.1035 (3)	0.0405 (10)	
C2	−0.03227 (17)	0.3163 (3)	0.1127 (3)	0.0390 (10)	
C3	0.01920 (16)	0.3715 (3)	0.1196 (2)	0.0374 (10)	
C4	0.01378 (17)	0.4713 (3)	0.1244 (3)	0.0377 (10)	
C5	−0.03978 (18)	0.5125 (3)	0.1162 (3)	0.0401 (10)	
H5A	−0.0418	0.5794	0.1200	0.048*	
C6	−0.1360 (2)	0.3092 (3)	0.0963 (3)	0.0552 (13)	
H6A	−0.1710	0.3405	0.0887	0.066*	
C7	−0.1331 (2)	0.2110 (4)	0.1008 (4)	0.0736 (17)	
C8	−0.0802 (2)	0.1650 (4)	0.1120 (4)	0.0724 (17)	
C9	−0.0314 (2)	0.2151 (3)	0.1193 (3)	0.0582 (14)	
H9A	0.0033	0.1821	0.1287	0.070*	
C10	0.07331 (17)	0.3246 (3)	0.1239 (3)	0.0401 (10)	
C11	0.07575 (18)	0.2519 (3)	0.0643 (3)	0.0462 (11)	
C12	0.1791 (2)	0.2288 (4)	0.1375 (3)	0.0516 (12)	
C13	0.12424 (17)	0.3552 (3)	0.1849 (3)	0.0434 (11)	
C14	0.1290 (2)	0.1194 (4)	0.0207 (4)	0.0677 (15)	
H14A	0.1561	0.0711	0.0517	0.081*	
H14B	0.0909	0.0905	0.0041	0.081*	
C15	0.1443 (3)	0.1470 (4)	−0.0569 (4)	0.092 (2)	
H15A	0.1433	0.0907	−0.0915	0.138*	
H15B	0.1823	0.1743	−0.0409	0.138*	
H15C	0.1171	0.1938	−0.0886	0.138*	
C16	0.2289 (2)	0.3434 (5)	0.2500 (4)	0.0736 (17)	
H16A	0.2564	0.2910	0.2675	0.088*	
H16B	0.2200	0.3671	0.3001	0.088*	
C17	0.2549 (3)	0.4222 (6)	0.2130 (5)	0.107 (2)	
H17A	0.2893	0.4446	0.2545	0.161*	
H17B	0.2279	0.4746	0.1965	0.161*	
H17C	0.2643	0.3985	0.1639	0.161*	
C18	−0.14249 (17)	0.5130 (3)	0.0928 (3)	0.0437 (11)	
H18A	−0.1549	0.5188	0.1441	0.052*	
C19	−0.1571 (2)	0.5954 (4)	0.0329 (3)	0.0594 (14)	
H19A	−0.1768	0.6502	0.0484	0.071*	
H19B	−0.1302	0.6116	0.0016	0.071*	
C20	−0.1889 (2)	0.5045 (4)	0.0120 (3)	0.0610 (14)	
H20A	−0.1817	0.4647	−0.0324	0.073*	
H20B	−0.2283	0.5032	0.0144	0.073*	
C21	0.06135 (18)	0.5399 (3)	0.1271 (3)	0.0455 (11)	
C22	0.1124 (3)	0.6771 (5)	0.1981 (5)	0.094 (2)	
H22A	0.1302	0.6866	0.2587	0.113*	
H22B	0.1412	0.6496	0.1749	0.113*	
C23	0.0952 (3)	0.7671 (6)	0.1597 (6)	0.130 (3)	
H23A	0.0825	0.7578	0.0985	0.156*	
H23B	0.1288	0.8087	0.1728	0.156*	
C24	0.0490 (3)	0.8188 (5)	0.1840 (6)	0.112 (2)	
H24A	0.0610	0.8283	0.2452	0.134*	
H24B	0.0146	0.7789	0.1694	0.134*	
C25	0.0342 (4)	0.9178 (6)	0.1395 (6)	0.145 (4)	
H25A	−0.0032	0.9385	0.1429	0.174*	
H25B	0.0314	0.9106	0.0798	0.174*	

### Atomic displacement parameters (Å^2^)

**Table:**

	*U*^11^	*U*^22^	*U*^33^	*U*^12^	*U*^13^	*U*^23^
S1	0.0580 (9)	0.1077 (14)	0.1167 (15)	0.0380 (9)	0.0255 (9)	−0.0067 (10)
Cl1	0.0780 (11)	0.0645 (11)	0.284 (3)	−0.0280 (9)	0.0751 (15)	−0.0082 (14)
Cl2	0.1264 (17)	0.0849 (14)	0.161 (2)	−0.0058 (12)	0.0313 (15)	0.0051 (12)
F1	0.094 (3)	0.0357 (18)	0.222 (5)	−0.0059 (17)	0.067 (3)	0.003 (2)
O1	0.052 (2)	0.082 (3)	0.072 (3)	0.0024 (19)	0.0056 (19)	−0.036 (2)
O2	0.0491 (19)	0.075 (2)	0.043 (2)	0.0087 (17)	0.0004 (16)	−0.0185 (17)
O3	0.0477 (18)	0.070 (2)	0.060 (2)	−0.0072 (17)	0.0225 (17)	−0.0007 (17)
O4	0.0503 (19)	0.046 (2)	0.090 (3)	−0.0131 (16)	0.0254 (19)	−0.0231 (18)
N1	0.0313 (18)	0.036 (2)	0.045 (2)	0.0007 (16)	0.0143 (16)	−0.0002 (16)
N2	0.048 (2)	0.042 (2)	0.061 (3)	0.0062 (18)	0.027 (2)	−0.0024 (18)
N3	0.035 (2)	0.073 (3)	0.047 (2)	0.0094 (19)	0.0070 (18)	−0.008 (2)
C1	0.038 (2)	0.039 (3)	0.046 (3)	−0.003 (2)	0.017 (2)	−0.002 (2)
C2	0.038 (2)	0.037 (2)	0.043 (3)	0.003 (2)	0.012 (2)	−0.0028 (19)
C3	0.035 (2)	0.042 (3)	0.035 (2)	0.001 (2)	0.0089 (19)	−0.0015 (18)
C4	0.036 (2)	0.040 (3)	0.038 (3)	−0.001 (2)	0.0111 (19)	−0.0021 (18)
C5	0.043 (2)	0.037 (2)	0.042 (3)	−0.002 (2)	0.015 (2)	−0.0022 (19)
C6	0.042 (3)	0.046 (3)	0.080 (4)	−0.003 (2)	0.022 (3)	−0.003 (2)
C7	0.057 (3)	0.051 (3)	0.120 (5)	−0.016 (3)	0.036 (3)	−0.009 (3)
C8	0.065 (4)	0.036 (3)	0.126 (5)	0.000 (3)	0.044 (4)	0.000 (3)
C9	0.051 (3)	0.042 (3)	0.086 (4)	0.005 (2)	0.027 (3)	0.004 (2)
C10	0.038 (2)	0.041 (3)	0.041 (3)	0.003 (2)	0.012 (2)	−0.0051 (19)
C11	0.039 (2)	0.046 (3)	0.056 (3)	0.003 (2)	0.018 (2)	−0.005 (2)
C12	0.046 (3)	0.059 (3)	0.052 (3)	0.009 (2)	0.018 (2)	0.003 (2)
C13	0.037 (2)	0.056 (3)	0.037 (3)	0.005 (2)	0.011 (2)	−0.004 (2)
C14	0.069 (3)	0.049 (3)	0.092 (4)	−0.001 (3)	0.035 (3)	−0.021 (3)
C15	0.118 (5)	0.074 (4)	0.106 (5)	−0.016 (4)	0.066 (4)	−0.034 (4)
C16	0.044 (3)	0.108 (5)	0.057 (4)	0.010 (3)	−0.003 (3)	−0.010 (3)
C17	0.070 (4)	0.130 (6)	0.105 (6)	−0.020 (4)	0.001 (4)	−0.013 (5)
C18	0.035 (2)	0.050 (3)	0.049 (3)	0.008 (2)	0.017 (2)	0.006 (2)
C19	0.046 (3)	0.060 (3)	0.077 (4)	0.014 (3)	0.026 (3)	0.024 (3)
C20	0.038 (3)	0.073 (4)	0.071 (4)	0.010 (3)	0.014 (3)	0.005 (3)
C21	0.037 (2)	0.047 (3)	0.051 (3)	−0.001 (2)	0.011 (2)	0.000 (2)
C22	0.067 (4)	0.065 (4)	0.145 (7)	−0.019 (3)	0.023 (4)	−0.037 (4)
C23	0.068 (5)	0.105 (6)	0.203 (9)	−0.009 (5)	0.019 (5)	0.021 (6)
C24	0.088 (5)	0.091 (5)	0.150 (7)	−0.004 (4)	0.027 (5)	−0.002 (5)
C25	0.119 (7)	0.102 (6)	0.170 (9)	−0.009 (5)	−0.022 (6)	−0.002 (6)

### Geometric parameters (Å, º)

**Table:**

S1—C12	1.665 (5)	C10—C11	1.418 (6)
Cl1—C7	1.717 (5)	C14—C15	1.481 (8)
Cl2—C25	1.725 (7)	C14—H14A	0.9700
F1—C8	1.341 (6)	C14—H14B	0.9700
O1—C11	1.231 (5)	C15—H15A	0.9600
O2—C13	1.229 (5)	C15—H15B	0.9600
O3—C21	1.208 (5)	C15—H15C	0.9600
O4—C21	1.340 (5)	C16—C17	1.479 (8)
O4—C22	1.451 (6)	C16—H16A	0.9700
N1—C5	1.326 (5)	C16—H16B	0.9700
N1—C1	1.375 (5)	C17—H17A	0.9600
N1—C18	1.458 (5)	C17—H17B	0.9600
N2—C12	1.352 (6)	C17—H17C	0.9600
N2—C11	1.429 (5)	C18—C20	1.469 (6)
N2—C14	1.491 (6)	C18—C19	1.479 (6)
N3—C12	1.379 (6)	C18—H18A	0.9800
N3—C13	1.406 (5)	C19—C20	1.462 (7)
N3—C16	1.477 (6)	C19—H19A	0.9700
C1—C6	1.409 (6)	C19—H19B	0.9700
C1—C2	1.415 (5)	C20—H20A	0.9700
C2—C9	1.404 (6)	C20—H20B	0.9700
C2—C3	1.441 (5)	C22—C23	1.402 (9)
C3—C4	1.391 (6)	C22—H22A	0.9700
C3—C10	1.449 (5)	C22—H22B	0.9700
C4—C5	1.390 (5)	C23—C24	1.484 (10)
C4—C21	1.486 (6)	C23—H23A	0.9700
C5—H5A	0.9300	C23—H23B	0.9700
C6—C7	1.361 (7)	C24—C25	1.544 (10)
C6—H6A	0.9300	C24—H24A	0.9700
C7—C8	1.396 (7)	C24—H24B	0.9700
C8—C9	1.348 (7)	C25—H25A	0.9700
C9—H9A	0.9300	C25—H25B	0.9700
C10—C13	1.406 (6)		
			
C21—O4—C22	116.5 (4)	H15A—C15—H15C	109.5
C5—N1—C1	118.9 (3)	H15B—C15—H15C	109.5
C5—N1—C18	119.8 (3)	N3—C16—C17	111.4 (5)
C1—N1—C18	121.3 (3)	N3—C16—H16A	109.4
C12—N2—C11	124.5 (4)	C17—C16—H16A	109.4
C12—N2—C14	120.0 (4)	N3—C16—H16B	109.4
C11—N2—C14	115.5 (4)	C17—C16—H16B	109.4
C12—N3—C13	124.3 (4)	H16A—C16—H16B	108.0
C12—N3—C16	119.1 (4)	C16—C17—H17A	109.5
C13—N3—C16	116.6 (4)	C16—C17—H17B	109.5
N1—C1—C6	119.4 (4)	H17A—C17—H17B	109.5
N1—C1—C2	119.8 (4)	C16—C17—H17C	109.5
C6—C1—C2	120.8 (4)	H17A—C17—H17C	109.5
C9—C2—C1	117.2 (4)	H17B—C17—H17C	109.5
C9—C2—C3	122.0 (4)	N1—C18—C20	119.5 (4)
C1—C2—C3	120.8 (4)	N1—C18—C19	118.6 (4)
C4—C3—C2	115.8 (3)	C20—C18—C19	59.5 (3)
C4—C3—C10	122.8 (4)	N1—C18—H18A	115.9
C2—C3—C10	121.3 (4)	C20—C18—H18A	115.9
C5—C4—C3	120.2 (4)	C19—C18—H18A	115.9
C5—C4—C21	116.0 (4)	C20—C19—C18	59.9 (3)
C3—C4—C21	123.3 (4)	C20—C19—H19A	117.8
N1—C5—C4	124.1 (4)	C18—C19—H19A	117.8
N1—C5—H5A	117.9	C20—C19—H19B	117.8
C4—C5—H5A	117.9	C18—C19—H19B	117.8
C7—C6—C1	119.6 (4)	H19A—C19—H19B	114.9
C7—C6—H6A	120.2	C19—C20—C18	60.6 (3)
C1—C6—H6A	120.2	C19—C20—H20A	117.7
C6—C7—C8	119.6 (5)	C18—C20—H20A	117.7
C6—C7—Cl1	120.5 (4)	C19—C20—H20B	117.7
C8—C7—Cl1	119.9 (4)	C18—C20—H20B	117.7
F1—C8—C9	119.8 (5)	H20A—C20—H20B	114.8
F1—C8—C7	118.3 (5)	O3—C21—O4	125.1 (4)
C9—C8—C7	121.9 (5)	O3—C21—C4	123.4 (4)
C8—C9—C2	120.9 (4)	O4—C21—C4	111.3 (4)
C8—C9—H9A	119.6	C23—C22—O4	113.5 (6)
C2—C9—H9A	119.6	C23—C22—H22A	108.9
C13—C10—C11	119.8 (4)	O4—C22—H22A	108.9
C13—C10—C3	119.5 (4)	C23—C22—H22B	108.9
C11—C10—C3	120.5 (4)	O4—C22—H22B	108.9
O1—C11—C10	125.3 (4)	H22A—C22—H22B	107.7
O1—C11—N2	118.0 (4)	C22—C23—C24	117.0 (8)
C10—C11—N2	116.8 (4)	C22—C23—H23A	108.1
N2—C12—N3	116.2 (4)	C24—C23—H23A	108.1
N2—C12—S1	122.2 (4)	C22—C23—H23B	108.1
N3—C12—S1	121.7 (4)	C24—C23—H23B	108.1
O2—C13—N3	117.9 (4)	H23A—C23—H23B	107.3
O2—C13—C10	124.4 (4)	C23—C24—C25	112.8 (7)
N3—C13—C10	117.8 (4)	C23—C24—H24A	109.0
C15—C14—N2	112.8 (4)	C25—C24—H24A	109.0
C15—C14—H14A	109.0	C23—C24—H24B	109.0
N2—C14—H14A	109.0	C25—C24—H24B	109.0
C15—C14—H14B	109.0	H24A—C24—H24B	107.8
N2—C14—H14B	109.0	C24—C25—Cl2	113.3 (6)
H14A—C14—H14B	107.8	C24—C25—H25A	108.9
C14—C15—H15A	109.5	Cl2—C25—H25A	108.9
C14—C15—H15B	109.5	C24—C25—H25B	108.9
H15A—C15—H15B	109.5	Cl2—C25—H25B	108.9
C14—C15—H15C	109.5	H25A—C25—H25B	107.7
			
C5—N1—C1—C6	175.2 (4)	C12—N2—C11—O1	171.1 (5)
C18—N1—C1—C6	−2.4 (6)	C14—N2—C11—O1	−8.3 (6)
C5—N1—C1—C2	−3.3 (6)	C12—N2—C11—C10	−7.7 (6)
C18—N1—C1—C2	179.1 (4)	C14—N2—C11—C10	173.0 (4)
N1—C1—C2—C9	175.0 (4)	C11—N2—C12—N3	1.1 (7)
C6—C1—C2—C9	−3.5 (6)	C14—N2—C12—N3	−179.6 (4)
N1—C1—C2—C3	−1.8 (6)	C11—N2—C12—S1	−177.9 (3)
C6—C1—C2—C3	179.7 (4)	C14—N2—C12—S1	1.4 (6)
C9—C2—C3—C4	−171.0 (4)	C13—N3—C12—N2	4.1 (7)
C1—C2—C3—C4	5.6 (6)	C16—N3—C12—N2	−175.3 (4)
C9—C2—C3—C10	7.2 (6)	C13—N3—C12—S1	−176.8 (4)
C1—C2—C3—C10	−176.3 (4)	C16—N3—C12—S1	3.7 (7)
C2—C3—C4—C5	−4.6 (6)	C12—N3—C13—O2	177.8 (4)
C10—C3—C4—C5	177.3 (4)	C16—N3—C13—O2	−2.7 (6)
C2—C3—C4—C21	−176.7 (4)	C12—N3—C13—C10	−2.3 (7)
C10—C3—C4—C21	5.2 (6)	C16—N3—C13—C10	177.1 (4)
C1—N1—C5—C4	4.5 (6)	C11—C10—C13—O2	175.2 (4)
C18—N1—C5—C4	−177.9 (4)	C3—C10—C13—O2	−1.3 (7)
C3—C4—C5—N1	−0.3 (6)	C11—C10—C13—N3	−4.7 (6)
C21—C4—C5—N1	172.3 (4)	C3—C10—C13—N3	178.8 (4)
N1—C1—C6—C7	−176.7 (5)	C12—N2—C14—C15	−87.5 (6)
C2—C1—C6—C7	1.8 (7)	C11—N2—C14—C15	91.9 (6)
C1—C6—C7—C8	−0.2 (9)	C12—N3—C16—C17	92.7 (6)
C1—C6—C7—Cl1	178.2 (4)	C13—N3—C16—C17	−86.8 (6)
C6—C7—C8—F1	179.6 (5)	C5—N1—C18—C20	117.2 (5)
Cl1—C7—C8—F1	1.2 (8)	C1—N1—C18—C20	−65.2 (5)
C6—C7—C8—C9	0.5 (10)	C5—N1—C18—C19	48.1 (6)
Cl1—C7—C8—C9	−178.0 (5)	C1—N1—C18—C19	−134.3 (5)
F1—C8—C9—C2	178.6 (5)	N1—C18—C19—C20	109.3 (5)
C7—C8—C9—C2	−2.3 (9)	N1—C18—C20—C19	−107.7 (4)
C1—C2—C9—C8	3.8 (7)	C22—O4—C21—O3	−8.1 (7)
C3—C2—C9—C8	−179.5 (5)	C22—O4—C21—C4	176.5 (4)
C4—C3—C10—C13	43.7 (6)	C5—C4—C21—O3	−123.8 (5)
C2—C3—C10—C13	−134.2 (4)	C3—C4—C21—O3	48.6 (7)
C4—C3—C10—C11	−132.7 (5)	C5—C4—C21—O4	51.7 (5)
C2—C3—C10—C11	49.3 (6)	C3—C4—C21—O4	−135.9 (4)
C13—C10—C11—O1	−169.4 (5)	C21—O4—C22—C23	102.8 (7)
C3—C10—C11—O1	7.0 (7)	O4—C22—C23—C24	55.7 (10)
C13—C10—C11—N2	9.3 (6)	C22—C23—C24—C25	178.9 (7)
C3—C10—C11—N2	−174.3 (4)	C23—C24—C25—Cl2	−76.6 (9)

### Hydrogen-bond geometry (Å, º)

**Table:**

*D*—H···*A*	*D*—H	H···*A*	*D*···*A*	*D*—H···*A*
C18—H18*A*···O2^i^	0.98	2.32	3.014 (6)	127
C20—H20*A*···O3^ii^	0.97	2.56	3.233 (6)	126
C25—H25*A*···F1^iii^	0.97	2.51	3.371 (10)	148

Symmetry codes: (i) −*x*, *y*, −*z*+1/2; (ii) −*x*, −*y*+1, −*z*; (iii) *x*, *y*+1, *z*.
